# Pulmonary alveolar proteinosis in children on La Réunion Island: a new inherited disorder?

**DOI:** 10.1186/1750-1172-9-85

**Published:** 2014-06-14

**Authors:** Laurent Enaud, Alice Hadchouel, Aurore Coulomb, Laureline Berteloot, Florence Lacaille, Liliane Boccon-Gibod, Vincent Boulay, Françoise Darcel, Matthias Griese, Mélinée Linard, Malek Louha, Michel Renouil, Jean-Pierre Rivière, Bruno Toupance, Virginie Verkarre, Christophe Delacourt, Jacques de Blic

**Affiliations:** 1 CHU Sud Réunion, service de Pédiatrie, Saint-Pierre, La Réunion, France; 2 Service de Pneumologie Pédiatrique, AP-HP, Hôpital Necker-Enfants Malades, service de Pneumologie Pédiatrique, Centre de Référence pour les Maladies Respiratoires Rares de l’Enfant, 149-161 rue de Sèvres, 75015 Paris, France; 3 Université Paris-Descartes, Paris, France; 4 AP-HP, CHU Paris-Est Hôpital d’Enfants Armand Trousseau, service d’Anatomie et Cytologie Pathologiques, Paris, France; 5 AP-HP, Hôpital Necker-Enfants Malades, service de Radiologie Pédiatrique, Paris, France; 6 AP-HP, Hôpital Necker-Enfants Malades, service de Gastroentérologie Pédiatrique, Paris, France; 7 CHU Sud Réunion, service de Pneumologie, Saint-Pierre, La Réunion, France; 8 CHU Sud Réunion, service de Neurologie, Saint-Pierre, La Réunion, France; 9 Hauner Children’s University Hospital, Ludwig-Maximilians-University, Member of the German Center for Lung Research, Munich, Germany; 10 CHU Sud Réunion, Service de Radiologie, Saint-Pierre, La Réunion, France; 11 AP-HP, CHU Paris-Est Hôpital d’Enfants Armand Trousseau, laboratoire de Biochimie et Biologie Moléculaire, Paris, France; 12 CHU Nord Réunion, service d’Anatomie et Cytologie Pathologiques, Saint-Denis, La Réunion, France; 13 Eco-Anthropologie et Ethnobiologie, UMR 7206 CNRS, MNHN, Univ Paris Diderot, Sorbonne Paris Cité, Paris, France; 14 AP-HP, Hôpital Necker-Enfants Malades, service d’Anatomie et Cytologie Pathologiques, Paris, France

**Keywords:** Pulmonary alveolar proteinosis, Pulmonary fibrosis, Child, Liver disease

## Abstract

**Background:**

Pulmonary alveolar proteinosis (PAP) is very rare in children. Only a few small series have been published, with little information about long-term progression. The objective of our study was to describe the clinical, radiological and pathological features, and the long-term course of PAP in a cohort of 34 children from La Réunion Island.

**Methods:**

Data were retrospectively collected from medical files. Radiological and pathological elements were reviewed by two pediatric radiologists and three pathologists, respectively.

**Results:**

Thirteen cases were familial and 32/34 (94%) cases were family connected. Disease onset occurred in the first six months of life in 82% of the patients. Thoracic computed tomography scans showed the typical “crazy-paving” pattern in 94% of cases. Respiratory disease was associated with a liver disorder, with the detection of liver enlargement at diagnosis in 56% of cases. The course of the disease was characterized by frequent progression to chronic respiratory insufficiency, accompanied by the appearance of cholesterol granulomas and pulmonary fibrosis. Overall prognosis was poor, with a mortality of 59% and an overall five-year survival rate from birth of 64%. Whole-lung lavages were performed in 21 patients, with no significant effect on survival. Liver disease progressed to cirrhosis in 18% of children, with no severe complication.

**Conclusions:**

PAP in children from la Réunion Island is characterized by an early onset, associated liver involvement, poor prognosis and frequent progression to lung fibrosis, despite whole-lung lavages treatment. The geographic clustering of patients and the detection of many familial links between most of the cases strongly suggest a genetic etiology, with an autosomal recessive mode of inheritance.

## Background

Pulmonary alveolar proteinosis (PAP) is a rare syndrome characterized by the alveolar accumulation of lipoproteinaceous material [1]. Diagnosis is suggested on computed tomography (CT) scans, showing a typical “crazy paving” appearance and alveolar consolidations [2]. It is confirmed by periodic acid-Schiff (PAS) staining of bronchoalveolar lavage fluid (BALF) or histological examinations of lung biopsy specimens [3]. Most of the reported cases occur in adults and are related to the presence of anti-granulocyte macrophage colony stimulating factor (GM-CSF) autoantibodies [4-6]. PAP is very rare in children, with only a few small series and case reports published to date. It may be primary or secondary to various diseases, including immune deficiencies, metabolic disorders and infections [7]. The mechanisms underlying primary PAP in children remain unknown, except for 15 published cases, for which mutations were found in the genes encoding the alpha and beta chains of the GM-CSF receptor [8-13]. The long-term prognosis of primary PAP in children remains also largely unknown. Whole-lung lavages (WLL) have been reported to provide short-term benefits in both adults [5] and children [10,14]. However, the long-term benefits of such treatment are unknown for children. The aim of this study was to describe the clinical, radiological and pathological features and the long-term course of PAP in a cohort of 34 patients from La Réunion Island.

## Methods

### Study design

We retrospectively reviewed the files of 34 children born between 1970 and 2012, seen at La Réunion Island University Hospital, with a diagnosis of PAP of unknown cause, confirmed by pathological analysis. Eighteen of these cases have been published before in other series but with only a brief description [7,14-21]. In order to search for known causes of PAP, immunological explorations allowed to exclude immune deficiencies in all patients. Chromatography of amino and organic acids in plasma and urine were also performed in all patients and their results excluded the diagnosis of lysinuric protein intolerance in all of them. GM-CSF autoantibody assays on serum and BALF were performed in 13 patients. Sequencing of *SFPTB, SFTPC, ABCA3, CSF2RA* and *CSF2RB* genes was performed for 17, 16, 10, 10 and 10 children, respectively. Two patients were heterozygous for the missense variant p.Arg167Gln of the *SFTPC* gene. This variant is reported as a single nucleotide polymorphism (SNP) in computer databases (dbSNP reference: rs34957318), its minor allele frequency was estimated at 9% among Réunion Island population [18] and this SNP was also found in healthy individuals without any respiratory disorder [18]. The results of genes sequencing were normal in the other patients. Radiological and pathological data were reviewed by two pediatric radiologists and three pathologists, respectively. This study was approved by the Institutional Review Board of the French Respiratory Society (CEPRO 2013–019).

### Genealogical studies

Family trees of patients were constructed by questioning parents on their family history at diagnosis assessment and retrieved form medical files. Furthermore, for the purpose of this retrospective study, and when parental consent was obtained, we reconstructed the thorough ascending genealogy of patients, retracing their ancestry, generation by generation, back in time. We identified the ancestors of the patients from published genealogies for Reunion Island for the years before 1935 [22,23], or directly from the civil registration records for subsequent years. These genealogies were stored in a computer database comprising 6,105 individuals. We developed a computer program to extract all genealogical paths linking at least two patients and to identify couples of common ancestors. We used Graphviz software [24] to visualize the resulting genealogical network as a marriage node graph [25] in which each couple is represented by a single symbol (octagon) rather than by two distinct individuals.

### Data collection

The clinical data included sex, age and type of first symptoms; age, clinical and biological features at diagnosis; mode of diagnosis; disease course and patient outcome. A history of failure to thrive and/or progressive dyspnea, sometimes associated with cough, vomiting or digital clubbing, were considered as first symptoms [26]. The radiologic lesions were classified according to the Fleischner Society’s Glossary of Terms for Thoracic Imaging [27]. The pathological review assessed the following lesions: PAP, cholesterol clefts, pulmonary interstitial and intra-alveolar cholesterol granulomas (PICG), other inflammatory lesions and fibrosis. A semi-quantitative grading scale was used to score each lesion, with 0 indicating absence; + indicating rare or mild lesions; ++ indicating moderate or localized lesions and +++ indicating diffuse lesions.

### Statistical analysis

Quantitative variables are presented as medians and interquartile ranges (IQR). Qualitative variables are expressed as percentages. Data were analyzed with Statview software (Abacus Concepts Inc, Piscataway, New Jersey), by analysis of variance, Fisher’s exact test, simple regression and logistic regression. Survival analyses were performed by the Kaplan-Meier method and group comparisons by logrank tests. *P <* 0.05 was considered statistically significant.

## Results

### Genealogical studies

**Figure 1:**
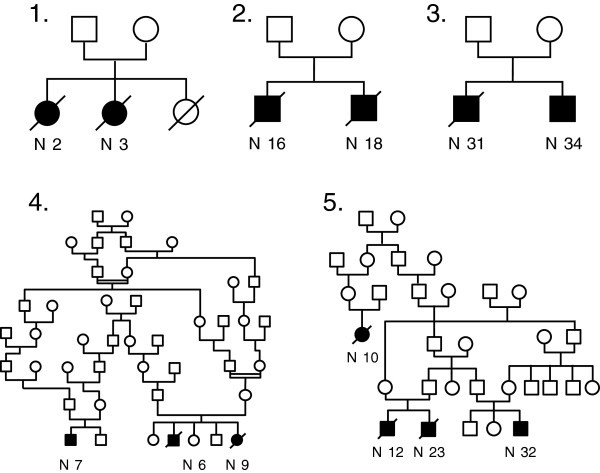
**Genealogical trees of familial cases of pulmonary alveolar proteinosis from La Réunion Island.** Genealogical trees of the 5 families with several cases of PAP. Familial forms of PAP in La Réunion Island in our study were characterized by the existence of at least one pair of affected siblings per family in addition of one or two cousins for pedigrees 4 and 5. The coexistence of affected and unaffected children among siblings, the fact that both sexes can be affected, the ascertained consanguinity in family 4 and the transversal distribution of the disease are highly suggestive of an autosomic recessive mode of inheritance.

**Figure 2:**
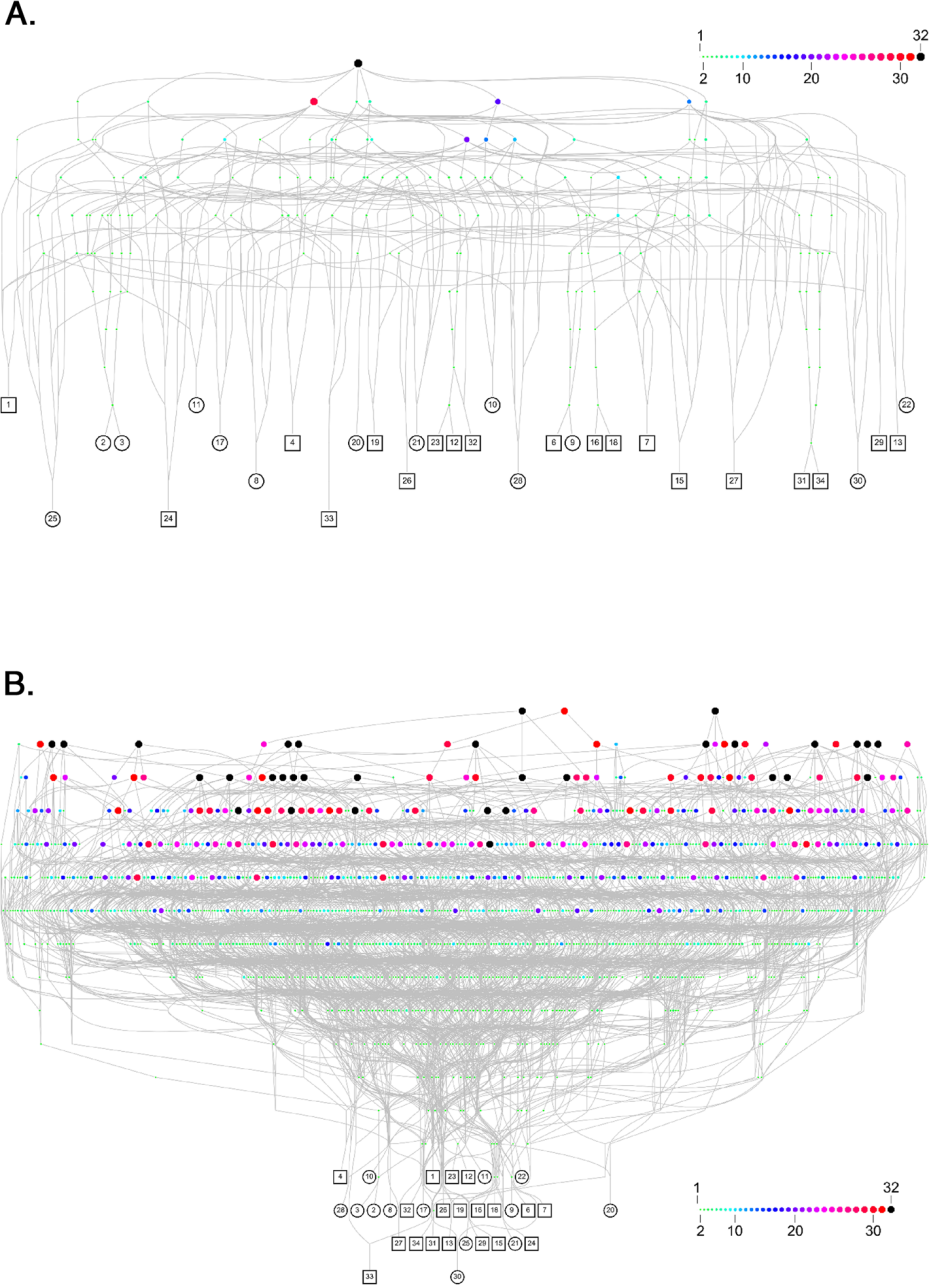
**Genealogical analysis of cases of pulmonary alveolar proteinosis from La Réunion Island. A**. Genealogical network connecting the 32 patients for which a parental consent was obtained to their most recent couple of common ancestors. Each patient (squares for boys and circles for girls) is denoted by his/her identifier. Each couple of ancestors is represented by an octagon of varying size and color according to the number of patients for which they are ancestors (from 1 to 32 as illustrated by the inner scale). The most recent couple of common ancestors (black octagon) married in 1731. Therefore, we can assert that all studied cases for which a consent was obtained are family connected. **B**. Deepest analysis connecting the 32 patients for which a parental consent was obtained to all their common ancestors. We found 32 couples of ancestors common to all patients (black octagons), each of them being a potential carrier of a genetic factor reponsible of the disease.

A primary analysis of families showed that 13 cases were familial and clustered in five families (Figure 1). Consent was obtained for thorough genealogical study for 25/27 families representing 32/34 patients (94% of our cohort). All of these patients share couples of common ancestors (Figure 2A and 2B). The most recent couple of common ancestors married in 1731. Going back further in time we found 32 couples of common ancestors to all studied patients, who are thus potential carriers of a genetic factor responsible for the disease (Figure 2B).

### Lung pathology findings at diagnosis

The diagnosis was confirmed by BALF analysis in 15 patients, by the examination of lung specimens in 18 patients, and by both methods in one patient.

The 16 diagnostic BAL were performed at a median age of 4.3 months (IQR: 3.4 – 13.7). In all reports, a classical “milky” appearance was reported and cytological analysis revealed the characteristic features of PAP, with large foamy macrophages engorged with PAS–positive inclusions and extracellular granular eosinophilic material, equally PAS-positive. Details on BAL cell counts and differentials are given in Table 1.

**Table 1:** Bronchoalveolar lavage cell counts and differentials at diagnosis

	**Median**	**IQR**
Total cell count (ml^-1^)*	620,000	330,000 – 900,000
*Differential (%)*		
Macrophages	62.5	48 – 75
Neutrophils	27.5	21.5 – 55
Lymphocytes	5	4.5 – 13

Data are presented as median and interquartiles range (IQR) for the 16 diagnostic broncho-alveolar lavages.

*Total cell count is reported for 8 BAL as BALF cellularity was undetermined (described as “uncountable”) for the 8 others.

**Figure 3:**
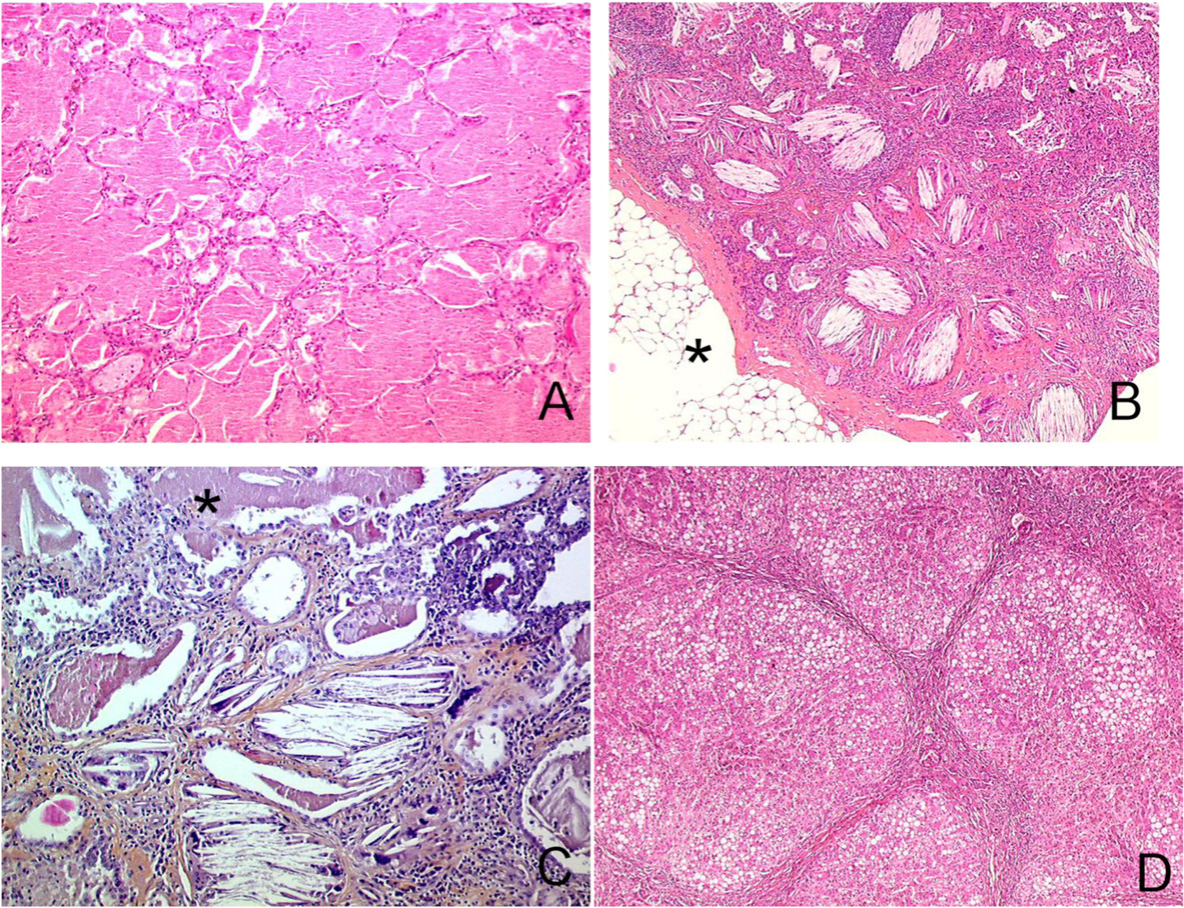
**Pathological features. A**: Lung histological specimen from a 9 months old boy, post-mortem examination. Diffuse intra alveolar accumulation of granular eosinophilic proteinaceous material with rare cholesterol clefts and normal interalveolar septa typical of PAP. There was no inflammation and no fibrosis. HES (×100). **B**: Lung histological specimen from a 9 years old girl, OLB. Numerous intra-alveolar cholesterol clefts with giant cells associated with inflammatory cells and fibrosis, typical of PICG. Lesions predominantly observed in the subpleural part of the lung parenchyma. Fatty involution of the visceral pleura (*). Tiny foci of PAP were also observed. HES (×100). **C**: Lung histological specimen from a 25 years old woman, post-mortem examination. Lung sample displayed both PAP on the upper part of the figure (*) and PICG with cholesterol clefts, giant cells granuloma, interstitial fibrosis and inflammation in the lower part of the figure. HES (×200). **D**: Liver histological specimen from a 2 years and 9 months old boy. Micro nodular cirrhosis, steatosis, ductular proliferation without active inflammation. HES (×100).

Examinations of lung specimens (*n =* 19) showed the presence of PAP lesions in variable amounts (Table 2). PAP lesions were isolated in five children (median age at lung sampling: 6.1 months, 5 – 11.8) (Figure 3A). PICG lesions were observed in eight patients, together with lesions of PAP and pulmonary fibrosis. These eight patients were the oldest at diagnosis (11.6 years, 8.3 – 15.4), and the greater abundance of PICG lesions than of PAP lesions even led to an initial diagnosis of cholesterol pneumonia in seven of them. In the other six children (7.1 months, 3.8 – 30), PAP lesions were observed together with small or moderate numbers of cholesterol clefts, without granuloma. One of these patients was also initially diagnosed with cholesterol pneumonia. Inflammatory aspects, including septal thickening and lymphocyte infiltrates, were present in 16 children, with no particular distribution according to age.

**Table 2:** Pathological features

		**Histological diagnosis specimen (*n =*** **19/34)**	**Last available histological specimen (*n =*** **14/34)**
**Age**		**30 months**	**7.3 years**
**median (IQR)**		**(5.3 months -10.3 years)**	**(6 years – 13.9 years)**
Mode: *n* (%)	OLB	16 (84)	1 (7)
	TBB	1 (5)	9 (65)
	Autopsy	2 (11)	2 (14)
	Native lung	0 (0)	2 (14)
Results: *n* (%)	PAP	19 (100)	13 (93)
	+	7 (37)	6 (43)
	++	3 (16)	5 (35)
	+++	9 (47)	2 (14)
	PICG	8 (42)	8 (57)
	+	0 (0)	1 (13)
	++	2 (25)	5 (62)
	+++	6 (75)	2 (25)
	Cholesterol clefts	14 (73)	14 (100)
	+	5 (36)	5 (36)
	++	3 (21)	4 (28)
	+++	6 (43)	5 (36)
	Fibrosis	8 (42)	10 (71)
	+	1 (13)	1 (10)
	++	2 (25)	3 (30)
	+++	5 (62)	6 (60)
	Inflammation	16 (84)	13 (93)
	+	9 (56)	9 (69)
	++	4 (25)	3 (23)
	+++	3 (19)	1 (8)

Definition of abbreviations: IQR = interquartile range; OLB = open lung biopsy; TBB = transbronchial biopsy; PAP = pulmonary alveolar proteinosis; PICG = pulmonary interstitial cholesterol granulomas. Data are presented at diagnosis and for the last available histological specimen. A semi-quantitative grading scale was used to score each lesion, with 0 indicating absence; + indicating rare or mild lesions; ++ indicating moderate or localized lesions and +++ indicating diffuse lesions. At diagnosis, PAP lesions were either isolated or associated with variable amounts of cholesterol clefts, cholesterol granulomas and fibrosis. Fibrosis was already observed at diagnosis in the 8 oldest cases of the series. At follow-up, although PAP lesions remained present but in a lesser extent, PICG and fibrosis lesions were more frequent.

### General characteristics and medical history

Twenty-two patients were boys (sex ratio = 1.8). Nine patients (26%) were born preterm and 25 (74%) presented intrauterine growth retardation, which was unexplained in 19 cases.

### Presentation at diagnosis: respiratory

Median age at diagnosis was 8.9 months (4–38.3). Age at diagnosis was significantly lower in boys (5 months (3.8 - 28.1) versus 19.4 months (6–148.2) in girls, *p =* 0.014). Respiratory symptoms and signs are described in Table 3. The interval between the onset of symptoms and diagnosis was variable, but early symptoms were observed in all but two children, with a median age at the onset of symptoms of 2.8 months (2 – 5). Twenty-eight patients (82%) presented with suggestive first symptoms within the first six months of life, with a severity that led to a rapid hospitalization for all of them. Four patients presented with first symptoms between the ages of seven and 15 months, whereas symptoms were not noted until the age of six years in the two remaining patients. Main features for each patient are described in the online Additional file 1: Table S1.

**Table 3:** Clinical and biological features at diagnosis

	*n*/total (%)*
*Clinical characteristics*	
Dyspnea and/or tachypnea	34/34 (100)
Cough	9/34 (26)
Low SaO_2_ (<92%) or low PaO_2_ (<70 mmHg)	17/34 (50)
Crackles at pulmonary auscultation^†^	15/34 (44)
Digital clubbing	13/34 (38)
Failure to thrive (weight < - 2 SD)	27/34 (79)
Anorexia	22/33 (67)
Vomiting	7/34 (21)
Hepatomegaly	19/34 (56)
Splenomegaly	10/34 (29)
*Biological characteristics*	
Anemia (Hb < 11 g/dL)	20/32 (63)
Hyperleukocytosis (≥15 000/mm^3^)	17/34 (50)
Thrombocytosis (≥450 000/mm^3^)	17/30 (57)
High IgG level	29/32 (90)
Hypoalbuminemia (<30 g/L)	17/25 (68)
Elevated AST	18/31 (58)
Elevated ALT	10/30 (33)
Elevated GGT	16/25 (64)
Elevated LDH	21/23 (91)

*: For some parameters, data were not available for all patients. ^†^: For the 19 others patients, auscultation was reported as normal.

**Figure 4:**
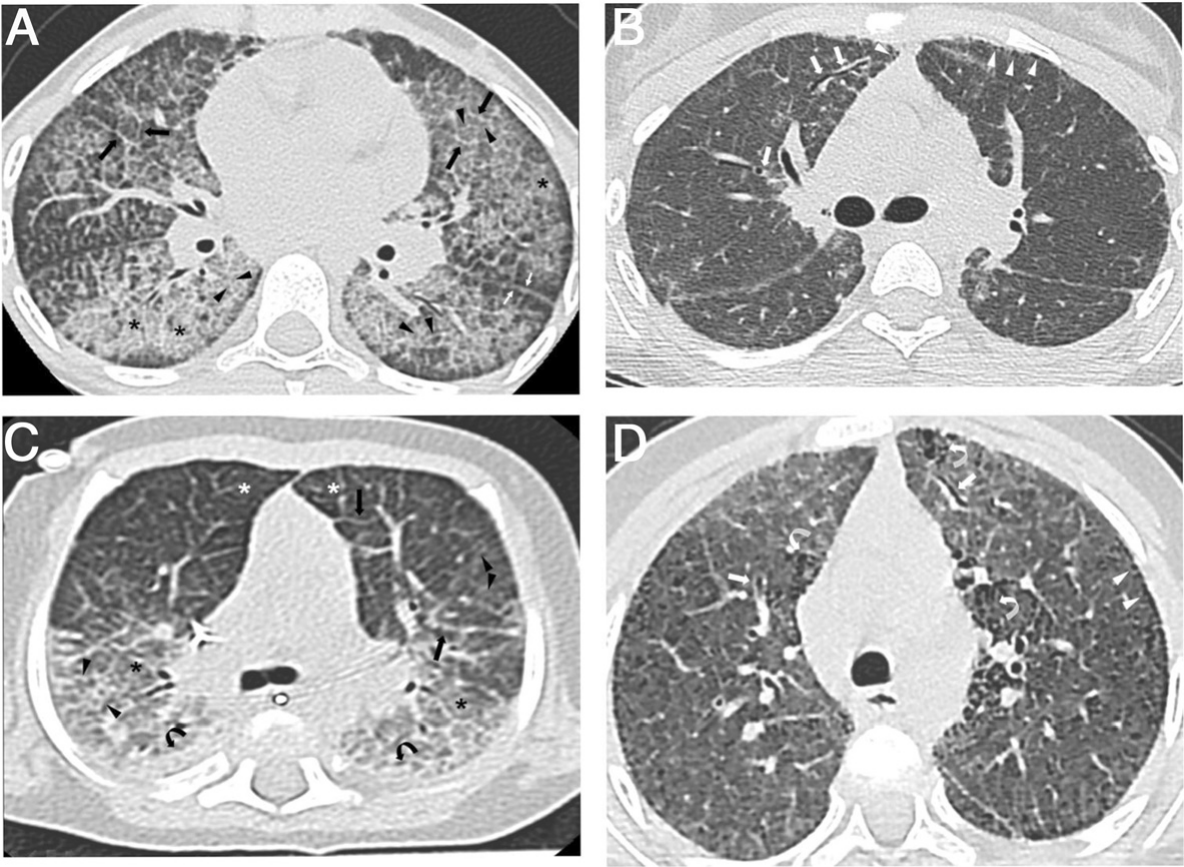
**Radiological features. A** and **B**: CT scan from a girl at the ages of 9 **(A)** and 13 **(B)** years old. **A**: Typical aspect of crazy paving pattern on thin axial sections: extensive areas of ground glass opacities (asterisks) superimposed with interlobular septa thickening (black arrows) and intralobular lines (black arrowheads). Irregular thickening of fissures are also noted (white arrows). **B**: Ground glass opacities, interlobular septa thickening and intralobular lines are less extensive than on the first CT scan while cystic lesions of a few millimetres (white arrowheads) are observed, with a subpleural distribution. Irregular traction bronchiectasis (white arrows) appeared that are initial signs of pulmonary fibrosis. **C** and **D**: CT scan of a girl at the ages of 8 months **(C)** and 5 years old **(D)**. **C**: This CT scan image shows a symmetric combination of extensive ground-glass opacities (asterisks), intralobular lines (arrowheads) and interlobular septa thickening (black arrows), associated to consolidation (black curved arrows) in posterior areas of the lungs. Note the airspace hyperinflation in the anterior compartment of the lung (white asterisks) responsible of an increasing density gradient from anterior to posterior areas. **D**: Extensive ground-glass opacities, intralobular lines and interlobular septa thickening are still present. Subpleural cystic lesions (white arrowheads) and signs of fibrosis: honey-combing (white curved arrows) and traction bronchiectasis (white arrows) appeared.

All patients had a chest X-ray during the diagnostic assessment. It showed an appearance of alveolo-interstitial or reticulo-nodular pattern in 31 patients and of “white lungs” in the other three patients. Thoracic CT scans taken at diagnosis or within the following year were available for 17 patients. The lesions observed are described in Table 4. Diffuse, symmetric involvement was typically observed, with a characteristic “crazy-paving” pattern in 94% of cases (Figure 4A). Consolidations were observed in 76% of cases, predominantly with a postero-basal location (Figure 4C). One patient with a very late diagnosis (first symptoms at 3 months and diagnosis at the age of 22 years) already had lesions suggestive of fibrosis at diagnosis, with traction bronchiectasis and honeycombing.

**Table 4:** Radiological features

	**Diagnosis CT**	**Last available CT**
	***n =*** **17**	***n =*** **13**
Age (median (IQR))	10 m (4.5 m – 22 y)	10 y (4.1 y – 14.1 y)
*Elementary lesions n (%)*		
Ground glass opacity	16 (94)	13 (100)
Consolidation	13 (76)	1 (8)
Interlobular septal thickening	17 (100)	13 (100)
Intralobular lines	17 (100)	13 (100)
Distension	13 (76)	1 (8)
Fissures thickening	16 (94)	13 (100)
Micronodules	1 (6)	0 (0)
Cystic lesions	1 (6)	12 (92)
Traction bronchiectasis	1 (6)	11 (85)
Honeycombing	1 (6)	5 (40)

Data are presented at diagnosis and for the last available CT scan. Observed lesions are reported according to the Fleischner Society’s Glossary of Terms for Thoracic Imaging (32). At diagnosis, ground glass opacities, interlobular thickening, intralobular lines and consolidations are the proeminent features. At last follow-up, consolidations have disappeared and fibrotic lesions are almost constant.

### Presentation at diagnosis: extra-respiratory

Extra-respiratory characteristics are detailed in Table 3. Failure to thrive was frequent and weight was equal to or below -4 standard deviations in 15 children. Liver involvement was also frequent, with a clinically enlarged liver noted in 19 of the 34 patients (56%). Abnormal liver test results were observed in 24 patients (71%). A liver ultrasound scan was carried out at diagnosis or within one year of diagnosis in 19 children and was abnormal in 17 cases (89%), with the liver found to be enlarged and containing a uniformly hyperechoic parenchyma suggestive of steatosis.

The importance of chronic inflammation was highlighted by high levels of immunoglobulin G and lactate dehydrogenase (LDH), thrombocytosis, hyperleukocytosis and anemia. Malnutrition with hypoalbuminemia was observed in 17 out of 25 patients (Table 3). No other organ involvement suggestive of a multisystem disease was detected.

### Respiratory course, treatment and outcome

#### Overall mortality and survival analyses

**Figure 5:**
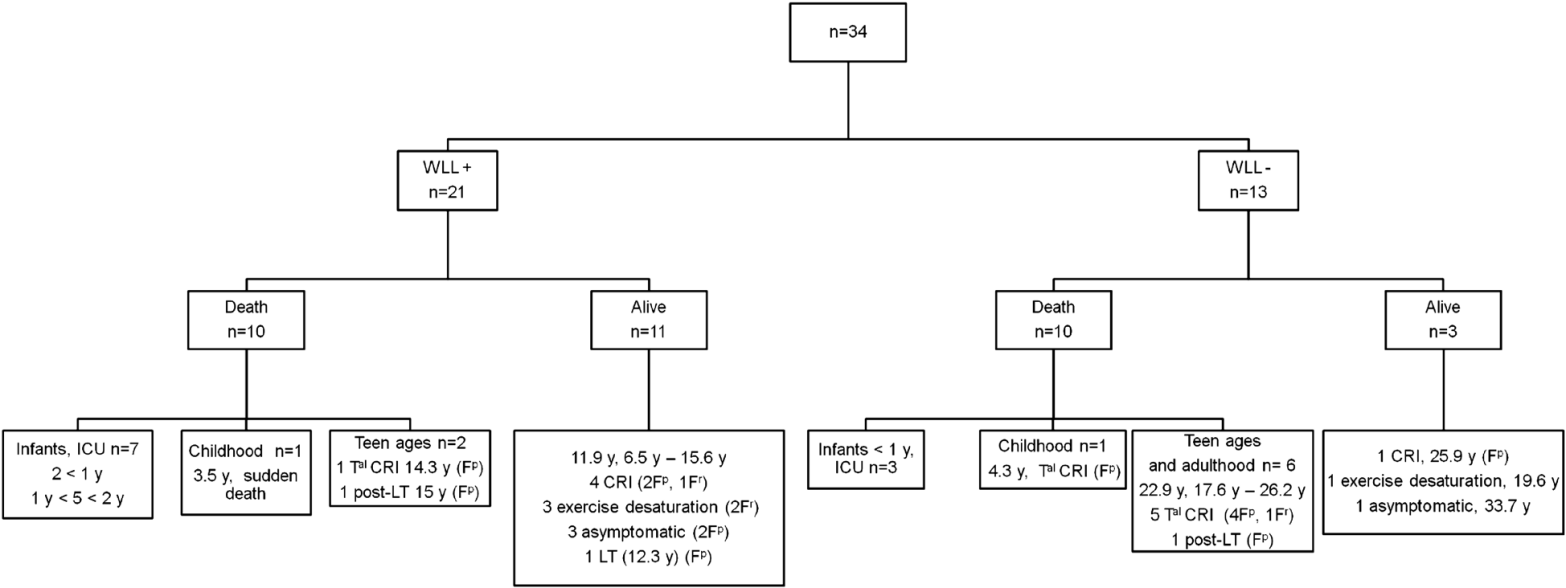
**Treatment and outcome.** Definition of abbreviations: WLL = whole lung lavages; ICU = intensive care unit; CRI = chronic respiratory insufficiency, T^al^ CRI = terminal chronic respiratory insufficiency; LT = lung transplantation; F^p^ = pathologically documented lung fibrosis; F^r^ = lesions suggestive of lung fibrosis on thoracic CT scan images; y = years. This figure consists in a flowchart of the study population according to treatment group. For group of patients, ages are expressed as median.

**Figure 6:**
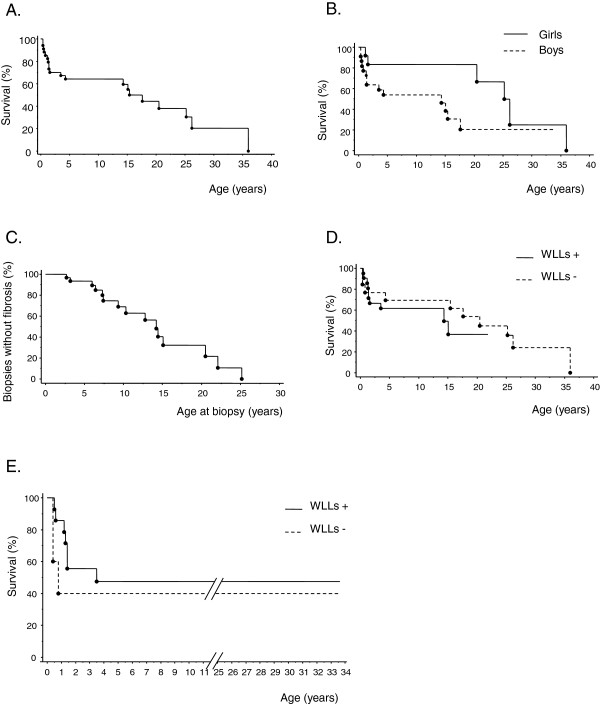
**Survival and occurrence of fibrosis.** All curves were performed using Kaplan-Meier method. **A**. Overall survival from birth showing a 5-year survival rate of 65%. **B**. Overall survival from birth according to gender. Five-year survival from birth was significantly lower in boys than in girls (54% in boys versus 83% in girls, logrank test, *p =* 0.04). **C**. Occurrence of fibrosis according to the age at which lung biopsy was performed showing a probability of significant pulmonary fibrosis of almost 50% by the age of 14 years old. **D**. Overall survival from birth with or without WLL. WLL did not significantly change global survival rates (logrank test, p = 0.46). **E**. Overall survival from birth with or without WLL in patients diagnosed before the age of one year with a larger number of WLL carried out earlier (WLL+, *n =* 14 / WLL-, *n =* 5). In this group, this treatment did not significantly change global survival rates (logrank test, p = 0.39).

Twenty patients (59%) died: five before the age of one year, five between the ages of one and two years, two later in childhood, four during adolescence and four during adulthood (Figure 5). Two major groups of patients can be identified (Figure 5): a group of patients dying early, within the first two years of life (infancy), in a context of rapidly progressive respiratory insufficiency (*n =* 10), and a group of children that survived infancy (*n =* 19) with subsequent progression, at various rates, to chronic respiratory symptoms, 9 of whom died. Besides, 4 patients are alive and asymptomatic at follow-up (ages: 5.3, 8.5, 16.5 and 33.7 years). One patient died suddenly after an unexplained malaise. The overall five-year survival rate from birth was 65% (Figure 6A). Age at death was significantly lower in boys (16.9 months (6.9 months – 14.2 years)) than in girls (22.8 years (1.6 – 26.2), *p =* 0.011)) and five-year survival from birth was also significantly lower in boys than in girls (54% versus 83%, *p =* 0.04; Figure 6B).

#### Progression to chronic respiratory insufficiency (CRI) and lung fibrosis

Nine patients died in a context of terminal CRI (Figure 5). Among these patients, two died from postoperative complications shortly after lung transplantation. Ten patients are still alive but have respiratory problems (median age: 12.5 years, 11 – 19.6): one of these patients requires continuous oxygen supplementation, four require nocturnal oxygen supplementation, four display exercise desaturation only and one underwent lung transplantation one year ago for terminal CRI. Since the lung transplantation, PAP did not recur in this patient. Indeed, she had several BAL and transbronchial lung biopsies after the transplantation and there was no sign of pulmonary alveolar proteinosis nor on BAL fluid neither on lung specimens’ examination.

Regarding lung function tests, lung volumes were normal in two patients. All the other patients in whom lung function tests were carried out displayed a restrictive pattern, with a median total lung capacity and median vital capacity equal to 47% (37% – 59%) (*n =* 10) and 42% (31% – 53%) (*n =* 15) of the predicted values, respectively. DL_CO_ values were below 80% of the predicted value in 15 of 18 patients.

Thoracic CT scans were available for review for 13 patients (Table 4). All images showed persistent thickening of the interlobular septa, intralobular lines and fissures (Figure 4B and D). In 12 of 13 patients, the course of the disease was characterized by the regression of consolidation and distention zones, associated with the appearance of lesions suggestive of fibrosis (Table 4, and Figure 4B and D) [27].

Histological progression was characterized by a progressive regression of PAP lesions accompanied by the appearance of cholesterol granulomas and fibrosis (Figure 3B, 3C and Table 2). At least one lung biopsy was carried out in 28 patients. Lung fibrosis lesions were identified on histological examination in 19 cases (69%). Kaplan-Meier analysis based on the occurrence of significant fibrosis (i.e. moderate or diffuse, *n =* 15 specimens) showed the probability of significant pulmonary fibrosis to be almost 50% by the age of 14 years (Figure 5C).

#### Disease course according to treatment group and the effects of WLL

Disease course according to treatment group is detailed in Figure 5. WLL were not performed in 13 patients for several reasons. Eight of these patients were the cases initially diagnosed with cholesterol pneumonia. All progressed to CRI and seven of them died. The other five cases were diagnosed before one year of age. Three died from respiratory insufficiency within the first year of life. PAP diagnosis in these cases was retrospective and based on post-mortem lung sample analysis. The other two patients were diagnosed before the introduction of WLL for children. Both are still alive and have reached adulthood. One has exercise dyspnea and the other is asymptomatic.

Overall, 67% (14/21) of the patients undergoing WLL and 77% (10/13) of those not undergoing this treatment survived infancy but this difference is not statistically significant (p = 0.7). Similarly, WLL seemed to have no influence on long-term prognosis. Progression to CRI and/or death occurred whether or not patients underwent WLL and this treatment had no significant effect on survival rates (Figure 6D, *p =* 0.46). However, it is difficult to determine the true effect of WLL due to the retrospective nature of this study and the heterogeneity of the treated and non-treated groups, particularly in terms of age at diagnosis. Even when we restricted the analysis to patients diagnosed before the age of one year with a larger number of WLL carried out earlier (WLL+, *n =* 14/WLL-, *n =* 5), WLL had no significant effect on overall survival rates (Figure 6E, *p =* 0.39).

#### Other treatments

When lung fibrosis occurred or when lung biopsies showed an important inflammation especially with lymphoid septal infiltrates, steroids were used either orally for the first reported cases or IV with high dose regimen from 1998. Other immunosuppressive treatments such as hydroxychloroquine, azathioprine or cyclophosphamide have been used occasionally. Details on medications and treatments are given in Table 5.

**Table 5:** Treatments

**Treatment**	**Patients n (%)**
WLL	21 (62)
Oral steroids	5 (15)
Intra-venous steroids	12 (35)
*Immunosuppressive medications*	
Hydroxychloroquine	1 (3)
Azathioprine	1 (3)
Cyclophosphamide	1 (3)
Mycophenolate mofetil	1 (3)

WLL, Whole lung lavages.

### Extra-respiratory outcome

A liver biopsy was performed in 12 children and two specimens were obtained from autopsy. The results were normal for 2 patients. However, one of these 2 patients presented high levels of GGT and a hyperechoic liver on ultrasound scan. Marked steatosis was observed in 9 cases. Fibrosis was observed in 10 patients and was classified as mild in 2 patients, extensive in 2 patients, and as cirrhosis in 6 patients (Figure 3D). In all children with extensive fibrosis or cirrhosis, regular screening was carried out for liver failure and portal hypertension. Grade I esophagal varices were found in five patients. Screening for hepatopulmonary syndrome was negative for all these patients. There were never signs of severe liver failure.

Among the patients that did not have a liver biopsy, 18 cases presented with hepatomegaly or abnormal liver tests results at some point in the course of the disease. At last-follow-up or at death, 11 patients had a persistent liver enlargement or abnormal liver tests results, 5 have normal liver tests results and no hepatomegaly and for 2 there were no complete available data for liver follow-up.

As a whole, 31 patients (91%) had a liver involvement at some point in the course of the disease.

Failure to thrive and oral feeding difficulties required initial parenteral nutrition in 10 patients (29%) and prolonged gastro-enteral feeding in 23 patients (67%).

### Correlation of clinical and biological data with pathological features

PICG lesions predominated in histological examinations at diagnosis for some patients. We therefore carried out additional analyses to determine whether the presence of such lesions was associated with a specific pattern of disease. The 28 patients that had had at least one lung biopsy were divided into two groups according to the presence of PICG lesions at some point in the course of the disease (*n =* 13) or the absence of such lesions (*n =* 15). We then assessed the association of these lesions with age at biopsy and with the main features of the disease: age at first symptoms, liver involvement, WLL treatment and outcome (dead or alive). Age at biopsy was the only feature significantly associated with cholesterol granulomas (mean age 10.8 years in the “PICG + ” group versus 4 years in the “PICG -” group, *p =* 0.001). This suggests that PICG lesions are part of the disease and that these lesions appear during its progression.

## Discussion

We report the largest series of cases of PAP in children from La Réunion Island. This cohort had particular characteristics: early onset, within the first months of life, a severe course with frequent progression to pulmonary fibrosis, associated liver involvement and more severe disease in boys than in girls.

The early onset of the disease seems to be highly specific to our series. PAP is a very rare cause of chronic lung disease in children, and is mostly diagnosed during adulthood. A pediatric onset has been reported for the forms due to *CSF2RA* and *CSF2RB* mutations, but at a wide range of ages. For *CSF2RA* mutations, reported ages at the onset of symptoms range from 1.5 to 9 years [9]. For *CSF2RB* mutations, onset was neonatal in two cases, occurred at the ages of one month and at nine years in two other cases and during adulthood for the last case [11-13].

The second characteristic feature of our series was the severity of the disease, with a high rate of mortality and frequent progression to pulmonary fibrosis. Published mortality data relate to adult forms and report a better prognosis, with a five-year survival rate from diagnosis of 88% [5]. Moreover, in adult PAP, and for autoimmune forms in particular, lung fibrosis is rarely described. Several case reports have mentioned focal interstitial fibrosis associated with adult PAP [28-32], but diffuse fibrosis is actually a very rare occurrence, with only a few cases reported [28,33-38]. In a cohort of 223 patients with autoimmune PAP, lung fibrosis occurred in only two cases (0.9%) [4]. In a retrospective radiologic study of 139 thoracic CT scans from 27 patients, substantial regions of fibrosis were observed in only two patients (7%) [2]. In the adult with a *CSF2RB* mutation, thoracic CT scans showed the progressive appearance of traction bronchiectasis and cystic lesions suggestive of fibrosis [12], but no lung fibrosis was described in children bearing *CSF2RA* mutations [9,10].

Given the small size of our population and the retrospective nature of the study, it is difficult to evaluate the efficacy of WLL in this cohort. Indeed, our clinical experience and management of patients diagnosed in the last 10 years suggest that WLL are effective in the short term, whereas statistical analyses on the entire cohort showed that there was no difference in survival between treated and untreated children. There are several possible reasons for this apparent discrepancy. First, the treated and untreated groups differed in terms of age at diagnosis, clinical presentation and severity. Second, this study covered a long time period (42 years), during which substantial changes occurred in both the WLL technique and the overall management of these patients. Prospective data collection would be required for the characterization of phenotypic groups and to identify the patients most likely to benefit from WLL.

Liver disease was also a distinctive feature in these patients, with no other organ involvement. As a whole, 31 patients (91%) had a liver involvement, and the absence of liver abnormalities in 3 patients could be attributable to a variable penetrance of this trait in this very likely genetic syndrome. Hepatic involvement can be observed in association with PAP in lysinuric protein intolerance [39] and adenosine deaminase deficiency [40,41], but has never been reported in other forms, particularly those of the autoimmune type that is the most reported type of PAP. In our cohort, it is debatable whether the steatosis observed arose due to the severe malnutrition often present at diagnosis. However, the ultrasound findings were at all ages and in all states of nutrition suggestive of fat overload in the liver. Liver disease progressed to extensive fibrosis or cirrhosis in seven children in the absence of severe complications of portal hypertension or liver failure.

The pathophysiological mechanism underlying PAP in these children remains unknown. The clustering of this disease in a specific geographic area, the existence of cases among closely related individuals and the results of our thorough genealogical study (94% of patients are family connected) strongly suggest a genetic origin. Moreover, the frequency of inbreeding in this geographic area during the 18^th^ and 19^th^ centuries [42], the absence of vertical transmission (i.e. parent-to-child transmission) with all parents being healthy, and the recurrence among siblings are highly suggestive of an autosomal recessive mode of inheritance.

As the GM-CSF pathway has been implicated in the autoimmune form and in known genetic forms, genes encoding targets or partners of GM-CSF (reviewed in [3] and [43]) are potentially good candidates. However, the available animal models of disrupted GM-CSF signalling [44,45] inducing a PAP phenotype have no liver phenotype and display no progression to pulmonary fibrosis. Furthermore, a dysfunction of GM-CSF signalling would led to alveolar macrophages’ dysfunction. In this hypothesis, one could expect that the disease would recur in transplanted patients. The absence of recurrence of the disease in the alive patient who underwent lung transplantation one year ago is one argument against the pivotal role of macrophages in the pathophysiology of this disorder. Nevertheless, it is impossible to draw definitive conclusions from the analysis of a single case with a relatively short follow-up since lung transplantation.

The associated liver involvement observed in our series raised questions about possible abnormal phospholipid metabolism or transport in both type II alveolar epithelial cells (type II AEC) and hepatocytes, potentially leading to abnormal surfactant turnover and fat overload in the liver. The fibrotic progression of the disease also suggests a potential role of type II AEC in this disease. Pathological review showed that inflammatory lesions were present at diagnosis in 16 children, seven of whom were investigated before the age of two years, suggesting that the inflammatory and subsequent fibrotic processes may occur at the same time as the accumulation of abnormal lipoproteinaceous material, rather than after this accumulation. PAP is classically characterized by preservation of the architecture of the alveoli, with normal and thin alveolar walls and rare descriptions of lymphocytic infiltrates [3,5]. In interstitial lung diseases caused by *SFTPC* mutations, mutant surfactant protein C has been shown to accumulate in the endoplasmic reticulum (ER), leading to ER stress, type II AEC injury, inflammation and the activation of apoptosis [46,47]. All these mechanisms seem to be involved in the fibrotic progression associated with *SFTPC* mutations [48]. In the disease described here, similar mechanisms could be suggested, with a genetic mutation leading to an abnormal protein that is misfolded in the ER, leading to inflammation and fibrosis. The defective functioning of this abnormal protein would also lead to abnormal surfactant turnover and accumulation in the alveoli. The important systemic inflammation in those children could also play a role in the development of lung fibrosis.

The reasons for the greater severity of disease in boys remain to be determined, and will probably become clearer when genetic analyses have determined the etiology of the disease. X-linked inheritance is very unlikely because both boys and girls can be affected by the disease which exclude a recessive X-linked disorder. As mentioned above, there is no case of parent-to-child transmission and this element *a priori* excludes a dominant X-linked mode of inheritance. Instead, modifier genes or hormonal factors may be involved.

In conclusion, we describe a cluster of 34 cases of primary PAP in children from La Réunion Island with very particular features, including a high rate of mortality and frequent progression to cholesterol granulomas and pulmonary fibrosis, despite WLL treatment. In this setting, the cause is very likely to be genetic, with an autosomal recessive mode of inheritance. Genetic studies should make it possible to determine the cause and mechanism of PAP and fibrosis in this population.

## Abbreviations

PAP: Pulmonary alveolar proteinosis; CT: Computed tomography; PAS: Periodic acid-Schiff; BALF: Broncho-alveolar lavage fluid; GM-CSF: Granulocyte macrophage colony stimulating factor; WLL: Whole lung lavages; SNP: Single nucleotide polymorphism; PICG: Pulmonary interstitial and intra-alveolar cholesterol granulomas; IQR: Interquartile ranges; CRI: Chronic respiratory insufficiency; AEC: Alveolar epithelial cells; ER: Endoplasmic reticulum.

## Competing interests

The authors declare that they have no competing interests.

## Authors' contributions

LE, AH, CD and JdB contributed to conception and design, and to all steps of the study. LE, AH, AC, LB, LBG, VB, FL, ML, ML, JPR, and VV. contributed to acquisition of data. LE, AH, AC, LB, LBG, FD, FL, ML, MR, BT, VV, CD and JdB contributed to analysis and interpretation of data. LE, AH, AC, LB, FL, CD and J.d.B have been involved in drafting the manuscript. LE, AH, AC, LB, FL, MG, MR, CD and JdB have been involved in revising critically the manuscript. All authors have given final approval of the version to be published.

## Supplementary Material

Additional file 1: Table S1 Individual characteristics of patients. Click here for file
