# Supplementary material for: Pulmonary alveolar proteinosis in children on La Réunion Island: a new inherited disorder?
Source: Orphanet J Rare Dis. 2014 Jun 14;9:85. doi: 10.1186/1750-1172-9-85 (PMC4062771; doi:10.1186/1750-1172-9-85)
Supplement: Additional file 1: Table S1 — Individual characteristics of patients. [file 1750-1172-9-85-S1.docx]

Additional file 1: Table 1: Individual characteristics of patients

| ID | Sex | age at symptoms onset (m) | age at PAP diagnosis | Clinical signs at diagnosis | diagnosis mode | liver disease | pathological pulmonary fibrosis | WLL | other treatment | outcome (dead or alive) | age at death | current age if alive | current outcome  if alive |
| --- | --- | --- | --- | --- | --- | --- | --- | --- | --- | --- | --- | --- | --- |
| 1 | M | 2 | 12.8 y | dyspnea, low SaO_2_, digital clubbing, FTT | biopsy | HMG | diffuse | no | no | dead | 17.6 y |  |  |
| 2 | F | 72 | 10.3 y | dyspnea, digital clubbing | biopsy | high AST and ALT | diffuse | no | oral steroids | dead | 36 y |  |  |
| 3 | F | 5 | 9.3 y | dyspnea, digital clubbing | biopsy | no | diffuse | no | oral steroids | dead | 26.2 y |  |  |
| 4 | M | 1 | 9.9 m | dyspnea, cough, low SaO_2_, FTT | biopsy | HMG | no | no | no | dead | 9.9 m |  |  |
| 5 | M | 7 | 3.2 y | dyspnea, digital clubbing, FTT | biopsy | no | diffuse | no | no | dead | 4.3 y |  |  |
| 6 | M | 1 | 3.8 m | dyspnea, cough, low SaO_2_, FTT | biopsy | HMG, high AST and ALT, steatosis | no | no | oral steroids | dead | 4.3 m |  |  |
| 7 | M | 3 | 5.3 m | dyspnea, cough, FTT | biopsy | HMG, high AST, ALT, GGT, steatosis mild fibrosis | no | no | no | alive |  | 32.7 y | asymptomatic |
| 8 | F | 1 | 14.4 y | dyspnea, digital clubbing, FTT | biopsy | HMG, high GGT | diffuse | no | IV steroids | dead | 20.5 y |  |  |
| 9 | F | 2 | 3.2 m | dyspnea, low SaO_2_, FTT | biopsy | HMG, high AST and ALT | no | yes | oral steroids | dead | 14.5 m |  |  |
| 10 | F | 72 | 16.3 y | dyspnea, digital clubbing | biopsy | high GGT, HMG | diffuse | no | oral steroids | dead | 25.2 y |  |  |
| 11 | F | 3 | 22.1 y | dyspnea, digital clubbing | biopsy | HMG, high GGT | moderate | no | no | alive |  | 24.9 y | CRI, nocturnal oxygen |
| 12 | M | 15 | 2.5 y | dyspnea, digital clubbing, FTT | biopsy | no | no | no | IV steroids | dead | 15.4 y |  |  |
| 13 | M | 3,5 | 7.9 m | dyspnea, low SaO_2_, FTT | both | HMG, high AST, ALT and GGT, steatosis extensive fibrosis | no | yes | no | dead | 16.5 m |  |  |
| 14 | M | 2,5 | 4.2 m | dyspnea, cough, low SaO_2_, FTT | BAL | HMG | undetermined | yes | no | alive |  | 20.9 y | exercise desaturation |
| 15 | M | 3 | 3.8 y | dyspnea, low SaO_2_, digital clubbing, FTT | biopsy | HMG, high AST | diffuse | yes | IV steroids mycophenolate mofetil | dead | 15.1 y |  |  |
| 16 | M | 2 | 4.6 m | dyspnea, low SaO_2_, FTT | biopsy | HMG, high AST and GGT | no | no | no | dead | 4.5 m |  |  |
| 17 | F | 2 | 6.1 m | dyspnea, low SaO_2_, FTT | biopsy | HMG, high AST, steatosis and mild fibrosis | diffuse | no | no | alive |  | 18.6 y | exercise desaturation |
| 18 | M | 3 | 3.5 m | dyspnea, low SaO_2_, FTT | BAL | HMG, high GGT steatosis | undetermined | yes | no | dead | 6.9 m |  |  |
| 19 | M | 3 | 7.4 y | dyspnea, cough, digital clubbing | biopsy | HMG, high AST, ALT and GGT | moderate | yes | IV steroids, hydroxychloroquine cyclophosphamide | dead | 14.3 y |  |  |
| 20 | M | 2 | 11.9 m | dyspnea, low SaO_2_, digital clubbing, FTT | BAL | HMG, high AST, ALT and GGT, cirrhosis | no | yes | no | alive |  | 15.8 y | asymptomatic |
| 21 | F | 9 | 16.1 m | dyspnea, low SaO_2_, FTT | BAL | HMG, high AST, ALT and GGT, | no | yes | IV steroids | alive |  | 14.9 y | exercise desaturation |
| 22 | F | 3 | 15.7 m | dyspnea, low SaO_2_, digital clubbing, FTT | biopsy | HMG, high AST, | no | yes | no | dead | 18.6 m |  |  |
| 23 | M | 2 | 3.7 m | dyspnea, FTT | BAL | HMG, high GGT, steatosis cirrhosis | undetermined | yes | no | dead | 15.5 m |  |  |
| 24 | M | 10 | 2.3 y | dyspnea, low SaO_2_, digital clubbing | BAL | high AST, HMG | no | yes | IV steroids | alive |  | 12 y | CRI, nocturnal oxygen |
| 25 | F | 2,5 | 5.9 m | dyspnea, cough, FTT | BAL | high AST, ALT and GGT, HMG | mild | yes | IV steroids | alive |  | 11.5 y | lung transplantation one year ago |
| 26 | M | 6 | 15.5 m | dyspnea | BAL | HMG, high AST, ALT and GGT | diffuse | yes | IV steroids | alive |  | 11.1 y | CRI, continuous oxygen |
| 27 | M | 3 | 4.2 m | dyspnea, low SaO_2_, FTT | biopsy | HMG, high AST, ALT and GGT, steatosis extensive fibrosis | no | yes | no | dead | 17.3 m |  |  |
| 28 | F | 6 | 22.7 m | dyspnea, cough, FTT | BAL | HMG | no | yes | IV steroids | alive |  | 10.2 y | exercise desaturation |
| 29 | M | 1 | 4 m | dyspnea, cough, low SaO_2_, FTT | BAL | HMG, high AST, ALT and GGT, steatosis cirrhosis | diffuse | yes | IV steroids | alive |  | 7.8 y | asymptomatic |
| 30 | F | 1,5 | 2.9 m | dyspnea, FTT | BAL | HMG, high AST and GGT, cirrhosis | mild | yes | IV steroids azathioprine | alive |  | 5.8 y | CRI, nocturnal oxygen |
| 31 | M | 1,5 | 3.2 m | dyspnea, FTT | BAL | HMG, high AST, ALT and GGT, steatosis cirrhosis | undetermined | yes | no | dead | 3.5 y |  |  |
| 32 | M | 1 | 1.8 m | dyspnea, FTT | BAL | HMG, high AST, ALT and GGT, cirrhosis | moderate | yes | no | alive |  | 4.6 y | asymptomatic |
| 33 | M | 1,5 | 4.4 m | dyspnea, cough, low SaO_2_, FTT | BAL | HMG, high AST and GGT, | undetermined | yes | IV steroids | dead | 5.4 m |  |  |
| 34 | M | 2 | 3 m | dyspnea, FTT | BAL | HMG, high AST and GGT, | undetermined | yes | no | alive |  | 1.1 y | CRI, nocturnal oxygen |

Abbreviations: M: male; F: female; SaO_2_: oxygen saturation; FTT: failure to thrive; CRI: chronic respiratory insufficiency; HMG: hepatomegaly; m: months; y: years; WLL: whole lung lavages; BAL: broncho-alveolar lavage; IV: intra-venous; AST: aspartate amino-transferase; ALT: alanine amino-transferase; GGT: gamma-glutamyl transferase.
